# Studying the functional conservation of cis-regulatory modules and their transcriptional output

**DOI:** 10.1186/1471-2105-9-220

**Published:** 2008-04-29

**Authors:** Denis C Bauer, Timothy L Bailey

**Affiliations:** 1 Institute for Molecular Bioscience, University of Queensland, Brisbane, Qld. 4072 Australia

## Abstract

**Background:**

*Cis*-regulatory modules (CRMs) are distinct, genomic regions surrounding the target gene that can independently activate the promoter to drive transcription. The activation of a CRM is controlled by the binding of a certain combination of transcription factors (TFs). It would be of great benefit if the transcriptional output mediated by a specific CRM could be predicted. Of equal benefit would be identifying *in silico *a specific CRM as the driver of the expression in a specific tissue or situation. We extend a recently developed biochemical modeling approach to manage both prediction tasks. Given a set of TFs, their protein concentrations, and the positions and binding strengths of each of the TFs in a putative CRM, the model predicts the transcriptional output of the gene. Our approach predicts the location of the regulating CRM by using predicted TF binding sites in regions near the gene as input to the model and searching for the region that yields a predicted transcription rate most closely matching the known rate.

**Results:**

Here we show the ability of the model on the example of one of the CRMs regulating the *eve *gene, MSE2. A model trained on the MSE2 in *D. melanogaster *was applied to the surrounding sequence of the *eve *gene in seven other *Drosophila *species. The model successfully predicts the correct MSE2 location and output in six out of eight *Drosophila *species we examine.

**Conclusion:**

The model is able to generalize from *D. melanogaster *to other *Drosophila *species and accurately predicts the location and transcriptional output of MSE2 in those species. However, we also show that the current model is not specific enough to function as a genome-wide CRM scanner, because it incorrectly predicts other genomic regions to be MSE2s.

## Background

Understanding transcriptional regulation is one of the main goals of the post-genomic era. Transcriptional regulation is a very complex biological phenomenon that is controlled by many factors [1]. Here, we focus on the transcriptional regulation mediated by discrete, genomic (DNA) regions called *cis*-regulatory modules (CRMs). Each CRM responds independently to a particular set of TFs, and causes a particular level of transcription to occur at the promoter it controls. CRMs might therefore be involved in "situation-specific" gene expression (e.g. tissue specificity). To understand what role CRMs are playing in "situation-specific" gene expression, we need computational models that predict the *genomic location *of the CRM responsible for triggering the expression of the target gene in a specific situation. Similarly important are computational models that predict the *transcription rate *mediated by a given DNA sequence. Those models can be applied to identify DNA regions with regulatory functions.

Here, we present a single computational model applicable to both prediction tasks on a specific gene in *D. melanogaster*. Our approach is distinct from previously reported CRM predictors (e.g. Berman *et al*. [2]) because it not only predicts the location of putative CRMs, but also assigns a specific transcriptional response to a single CRM. To accommodate the second prediction task, the model can also be used to predict the transcription rate of the target gene mediated by any DNA sequence.

To achieve this, we extend a recently-developed, steady-state, quantitative model of the transcriptional regulation of a particular gene in the fruit-fly (*D. melanogaster*) [9]. This model was originally described by Reinitz *et al*. [13], and will be referred to herein as the "Reinitz" model. Earlier work successfully tested the Reinitz model on the even-skipped gene (*eve*) in *D. melanogaster*, which controls segmentation in the fruit-fly and is expressed in seven stripes along the anteroposterior axis in the embryo [9]. More specifically, they applied the model to the CRM controlling the second (and to a lesser degree the seventh) stripe. This CRM is called "minimal stripe element two" (MSE2), and is located 1.7 kb upstream of the *eve *gene [15].

The ability of the Reinitz model to describe the output of MSE2 is of particular interest in light of a very recent study by Segal *et al*. [14]. They studied a different mathematical model of regulation in *D. melanogaster *that fails to predict the output of MSE2 correctly. One of the reasons that the Reinitz model accurately models the behavior of MSE2 might be that a different regulatory role was assigned to one of the TFs, Hunchback (hb). Segal *et al*. used hb as repressor while Janssens *et al*. used it as activator. Another difference between the approaches is that the Reinitz model was applied to a very detailed measurements of the output of MSE2 and the seven controlling TFs-hb, Bicoid (bcd), Caudal (cad), Giant (gt), Krüppel (kr), Knirps (kni) and Tailless (tll) [9,8]. This also sets the Reinitz model apart from other modeling approaches introduced by Bolouri *et al*. [5] and Zinzen *et al*. [17].

The Reinitz model aims to simulate the TF-DNA binding and interaction events. It divides the set of regulators into activators and repressors, here called "quenchers". The model contains adjustable parameters that describe the maximal transcription rate (*R*_0_) and the energy barrier (*X*_0_). In addition, the parameter set also contains two variables for each TF that define its ability to bind to the DNA (association constant, *K*) and its effectiveness as a quencher or activator, (*E*). The model predicts the transcriptional output of a promoter due to the action of a particular CRM, where the CRM is represented by a transcription factor binding site map ("TFBS-map") specifying the positions and binding strengths of all binding sites for the set of TFs. TFBS binding strengths are given as log-odds scores derived from a position weight matrix (PWM) for the corresponding TF.

Janssens *et al*. [9] first applied the Reinitz model to the MSE2 in *D. melanogaster*. They used a mixture of predicted and experimentally verified TFBSs, which we will refer to as "knowledge-based" TFBSs. They also introduced additional free parameters to the original Reinitz model that allow for individual binding site affinities that are not in agreement with the theoretical justification for the Reinitz model. We refer to this variation of the original model as the "Janssens model".

Janssens *et al*. [9] showed that the Janssens model could be trained to fit the data on the expression level of *eve *at different times and locations in the developing *D. melanogaster *embryo. They also showed that the model correctly predicts (qualitatively) the effect on *eve *expression in a *D. melanogaster *mutant lacking one TF (kni). In addition, they showed that the model correctly predicts qualitative changes in anteroposterior location of *eve *expression, as well as increases and decreases in expression, caused by various mutations of TFBSs.

Janssens *et al*. [9] primarily studied the ability of a variation of the Reinitz model to accurately *describe *transcriptional output of the *eve *gene in *D. melanogaster *as a function of the concentration of certain TFs and their predicted binding to a particular CRM. The current work, however, aims to use the original Reinitz model in the two *predictive *tasks stated above: to predict the transcriptional output of the eve MSE2 in *D. melanogaster*, and to predict the genomic location of MSE2 in *D. melanogaster *and other *Drosophila *species.

An additional objective of the current work is the verification of the generalization accuracy of the Janssens model. By generalization accuracy, we refer to how well the model captures the underlying mechanisms of transcriptional regulation, rather than just fitting the training data. We do this using standard cross-validation.

It is believed that the *eve *gene in other *Drosophila *species is subject to the same regulatory mechanisms as it is in *D. melanogaster*. While the function is conserved, the MSE2 sequence and the extent of the regulatory region has changed. Ludwig *et al*. [10] investigated *D. pseudoobscura *and three close relatives to *D. melanogaster*. They found that a DNA fragment containing the MSE2 from the other species was able to drive the correct stripe 2 expression when combined with a *lacZ *reporter and transfected into *D. melanogaster*.

To investigate if the Reinitz model correctly predicts the stripe 2 expression given the MSE2 regions from other species, we train the model on the MSE2 region in *D. melanogaster*, and evaluate its predictions on the MSE2 regions in seven other *Drosophila *species. The goal is to confirm the ability of the model to correctly predict the transcriptional output mediated by the CRM in other species. This will verify the assumption that, though the MSE2 region has evolved, the regulatory mechanisms have stayed the same [10].

To investigate the ability of our approach to identify the location of a specific CRM, we use it in a simple DNA scanning algorithm. The task is to identify the MSE2 region in the DNA sequence surrounding *eve *in *D. melanogaster *and other species of *Drosophila *by finding the DNA region that the model predicts will produce the correct transcriptional output. This will determine the specificity of the model in distinguishing between the true MSE2 and other TFBS-clusters with TFBSs of the same set of TFs but with "wrong" order, spacing and quantity.

The previous work by Janssens applied (a variant of) the Reinitz model to TFBS-maps with mainly experimentally-validated TFBSs. However, in general, only a small fraction of functional TFBSs are experimentally validated. Therefore, for the model more generally applicable in a scanning algorithm, it should be able to function well using TFBS-maps consisting solely of *predicted *TFBSs. However, because *in silico *prediction of TFBSs is notoriously error-prone [16], the model needs to be robust enough to cope with wrongly predicted and missing TFBSs. We investigate the robustness by comparing the performance of the Janssens model with the Reinitz model using predicted-only TFBS-maps for MSE2.

## Results

### 0.1 Assessing the generalization accuracy of the Janssens model

To assess the performance of the benchmark model-the Janssens model-from a machine learning point of view, we reproduce a modification of the experiment reported by Janssens *et al*. [9]. Rather than training on the full data set as done by Janssens *et al*. [9], we performed a seven fold cross-validation (CV) (see Sec. 0.9 in Methods). Briefly, the data consists of measurements taken at seven time points during the early development of the *D. melanogaster *embryo. We train the model on all data corresponding to six of the seven time points, and test on the remaining data. We repeat this seven times, leaving out each time point once. The accuracy of the model on each of the seven held-out data sets is a measure of its ability to generalize to unseen data. Because the training process is stochastic and not guaranteed to converge to the optimum parameter values, we repeat the training 30 times for each fold of cross-validation, starting the optimization from different randomly-selected parameter values each time.

**Figure 1:**
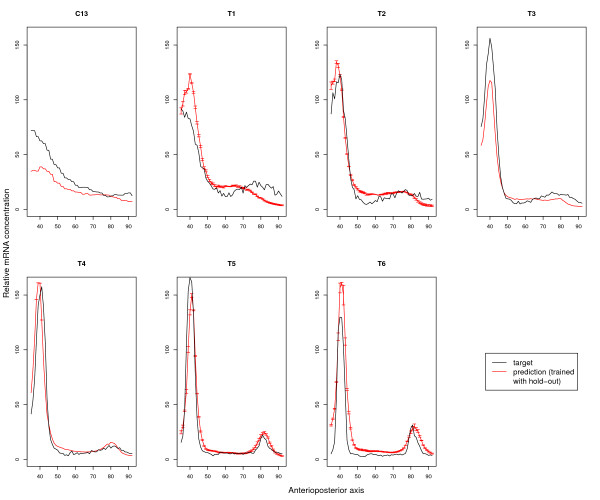
**Generalization accuracy of the Janssens model**. Each panel in the figure shows the measured mRNA level (black curve) and the model-predicted mRNA concentration (red) on the testing data for each of the cross-validation runs where the model was trained on all but this time point. Time points are indicated above each panel. The error-bars indicate the standard deviation among the 30 simulated annealing runs.

The Janssens model is able to fit the training data quite well, with an average (standard deviation) root-mean-squared (RMS) error over the six training time points, seven CV-folds and 30 repeats of 8.89 (0.34). This is an average error of 5.5% of the maximal observed mRNA expression rate. Though the testing (cross-validation) error is larger-12.95 (2.33)-, Fig. 1 shows that the characteristic expression of *eve *generated by MSE2 is *qualitatively *correctly predicted.

### 0.2 Using predicted-only TFBS-maps

In order to use the Reinitz model as part of a scanning algorithm capable of identifying DNA regions with regulatory function, it is desirable that the model is applicable to any DNA sequence. This means that the approach should be based on predicted TFBSs only. In this section we train the Reinitz model using predicted sites and compare its performance to the performance of the Janssens model. The TFBS prediction for our model is done as described in Methods Sec. 0.7, and results in 20 TFBSs compared to the 34 "knowledge-based" sites used by Janssens *et al*. [9].

Tab. 1 shows the comparison between the Janssens model and our model using predicted TFBSs. Again the reported RMS errors are an average over the six time points and 30 independent runs in each of the seven CV folds. The Janssens model fits the training data slightly better (RMS error 8.89 vs. 9.35). The difference in the training error is small, but statistically significant (two-tailed homoscedastic *t*-test *p*-value = 10^-16^). However, in terms of the generalization accuracy (as measured by testing error), the models are indistinguishable (two-tailed homoscedastic *t*-test *p*-value = 0.64). The better ability of the Janssens model to fit the training data might be due to the additional free parameters. These additional parameters appear to enable a closer fit to the training data, while not aiding in capturing the underlying mechanisms of transcriptional regulation, as evidenced by the statistically indistinguishable testing error of the two models.

**Table 1:** Comparison of the accuracy of the Janssens model and our model.

	*Cross validation*	
*Model*	*Training error (SD)*	*Testing error (SD)*

Janssens model	8.89 (0.34)	12.95 (2.33)
Model using predicted TFBS	9.38 (0.35)	13.06 (2.41)

The table shows the average (standard deviation) RMS error for three independent training runs using the Janssens model (knowledge-based TFBS map and extra free parameters) and our model (predicted-only TFBS map and no extra parameters), respectively. Training was done using the simulated annealing algorithm. For the model using predicted sites, we used a log-odds threshold of 9 bits and the *D. melanogaster *background base frequencies with a total pseudo-count of 1.5.

**Figure 2:**
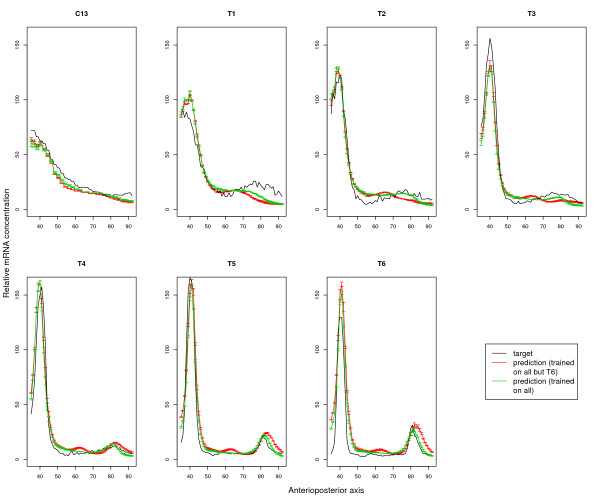
**Prediction output of TrainedAll model and TrainedMinusT6 model on all time-points**. Each panel in the figure shows the measured mRNA level (black curve) and the model-predicted mRNA concentration for one of the seven time points. The green curve belongs to TrainedMinusT6 model, which is trained on the redundancy reduced set and, red curve belongs to TrainedAll model, which is trained on all time-points. The error-bars indicate the standard deviation among the 30 simulated annealing runs.

The above experiment indicates that it should be possible to use the Reinitz model trained on predicted-only TFBSs (rather than the Janssens model) as part of a scanning algorithm. We therefore train a final model (TrainedAll model), trained on all time points. It achieves an average training error over the 30 independent repeats of 9.66 (0.01). We noticed that the data points from time points *T5 *and *T6 *are very similar to each other (See Sec. 0.9 in Methods). In order to avoid biasing the model, we also evaluate a second final model (TrainedMinusT6 model), which is trained on a "redundancy-reduced" training set, where all data from time point *T6 *is removed. This model has a training error of 9.00 (0.02). The output of both models is shown in Fig. 2 and the trained parameter values are given in the Supplementary material (Additional file 1).

### 0.3 Predicting the exact location of MSE2 in other *Drosophila *species

It is believed that the transcriptional output of *eve *mediated by MSE2 and the protein concentration of the seven TFs have stayed the same despite the speciation [10]. A good indication of the generality of the Reinitz model is therefore its ability to accurately predict the MSE2 location and its mediated transcriptional output in other *Drosophila *species. To test this, we apply TrainedAll model and TrainedMinusT6 model, trained on the MSE2 in *D. melanogaster*, to all windows in the 20 kb surrounding the *eve *gene in *D. melanogaster *and other *Drosophila *species (see Methods Sec. 0.11). We measure the RMS error for all time points by comparing the *predicted *output using the MSE2 in a given species with the *known *transcriptional output for *D. melanogaster*. Since we do not know the exact extent of the MSE2 in the other species we consider all window sizes ranging from 100 to 2000 bp (100 bp increment), and compute the RMS error of the prediction from each window compared with the known transcriptional output of *D. melanogaster *for all time points. The window with the lowest RMS error-the "optimal window"-is then the predicted MSE2. To measure the prediction accuracy, we calculate the performance coefficient (PC) [12] using the sequences aligned to MSE2 in *D. melanogaster *in the UCSC alignments [3] (see Sec. 0.10 in Methods) as the "gold standard" for the location and extent of MSE2 in the other species.

**Figure 3:**
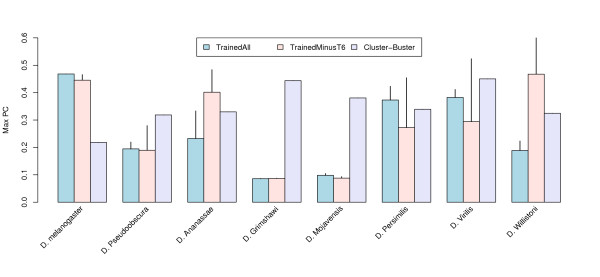
**Comparison of the performance of different methods for the prediction of the MSE2 locations in *D. melanogaster *and seven other *Drosophila *species**. The average PC for the 30 different TrainedAll models and TrainedMinusT6 models was calculated for different RMS thresholds. The best PC for each model is displayed in the figure along with the standard deviation. The maximal PC for cluster-buster was chosen among runs with different gap length between TFBSs within a cluster.

We compare the ability of our model to predict the location and extent of MSE2 in the various *Drosophila *species with the CLUSTER-BUSTER algorithm. CLUSTER-BUSTER searches a DNA sequence for clusters of matches to the set of motifs in its input. We provide CLUSTER-BUSTER with the same seven PWMs as used by our model. CLUSTER-BUSTER allows the user to set a parameter controlling the expected gap length between sites in a cluster. We ran CLUSTER-BUSTER with gap lengths from one to 29 and report the highest PC achieved in Fig. 3. To calculate PC for our method, an RMS threshold must be chosen (see Sec. 0.11). We compute the average PC of the 30 TrainedAll models and TrainedMinusT6 models using different RMS thresholds ranging from zero to 25, and report the highest PC achieved. As can be seen in Fig. 3, the predictive accuracy of our models is competitive or better than CLUSTER-BUSTER in three of the seven species other than *D. melanogaster *(on which the models were trained). On two distant species (*D. grimshawi *and *D. mojavensis*) both models are markedly inferior to CLUSTER-BUSTER. Surprisingly, the TrainedMinusT6 model does extremely well at predicting the location of MSE2 in the distantly related species *D. willistoni*. The results also indicate that the inclusion of time point T6 may be biasing the model toward the later time points. Although the TrainedMinusT6 model achieves a better maximum PC than TrainedAll model for only two species (*D. ananassae *and *D. willistoni*), the region with the lowest RMS error overlaps MSE2 on average in 5.5 species (averaged over the 30 TrainedMinusT6 models) compared with 3.3 species for the TrainedAll models (data not shown).

**Figure 4:**
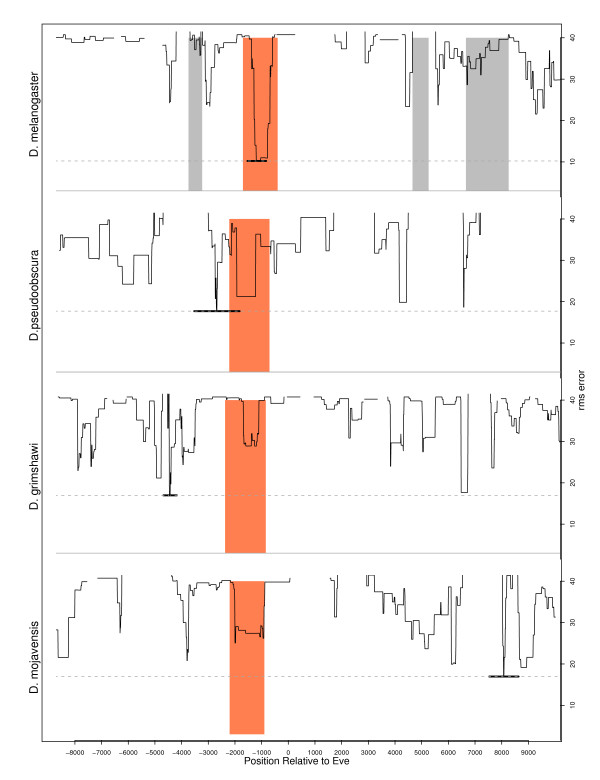
**Predicting MSE2 in four *Drosophila *species**. The figure shows the RMS distribution over the 20 kb surrounding *eve*, predicted by the best TrainedMinusT6 model, trained on the MSE2 region in *D. melanogaster*. The orange area shows the location of the annotated or homologous MSE2. The grey shaded areas show the location of other MSEs. The horizontal lines mark the location and extent of the window with the lowest error.

**Figure 5:**
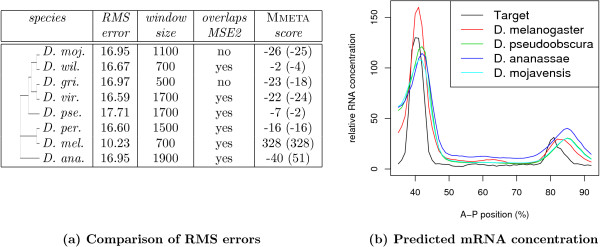
**Accuracy of the best TrainedMinusT6 model in predicting the location of MSE2 in other species**. The table shows the results of the model using predicted TFBSs trained on the 1300 bp MSE2 region from *D. melanogaster *as a predictor. Column one shows evolutionary distances of the examined species as a tree. Columns two to four shows the RMS error and window size of the optimal window and whether or not it overlaps with the UCSC-aligned MSE2. The last column shows the MMETA score, where the MSE2 from *D. melanogaster *is aligned with the optimal window (the UCSC-aligned MSE2). The abbreviations of the *Drosophila *species in column one are given in the Supplementary material.

The performance of our model is illustrated by the predictions made by the TrainedMinusT6 model with the best *training error *amongst the 30 repeats, herein referred to as "best TrainedMinusT6 model". Fig. 5a shows that the known (or UCSC aligned) MSE2 overlaps the best scoring window predicted by our best TrainedMinusT6 model in six out of eight species. For the two species where the optimal window does not overlap MSE2, the RMS errors at the MSE2 region are among the lowest as shown in Fig. 4. Also shown in the table is the prediction accuracy achieved by the optimal window over all time points.

To investigate if the predictive accuracy in the other *Drosophila *species is mainly due to the similarity of its MSE2 region to that of *D. melanogaster*, we compare the TFBS-map of the window with the best RMS error to the TFBS-map of the training window in *D. melanogaster *using the TFBS-map alignment tool MMETA [4]. Except for *D. melanogaster *itself, the MMETA scores are very low, indicating that only a small number of TFBSs in the two maps can be aligned. Therefore, the TFBS-maps in the regions predicted by the best TrainedMinusT6 model differ significantly from the MSE2 TFBS-map for *D. melanogaster*. The MMETA scores for the predicted MSE2 regions and the UCSC-aligned regions are almost the same (Fig. 5) in all but one species, indicating that the TFBS-map from the window predicted by TrainedMinusT6 model can be aligned to the TFBS-map from *D. melanogaster *with the same quality as the homologous MSE2 sequence identified by UCSC. Interestingly, in *D. ananassae *the homologous MSE2 sequence achieves a better MMETA score than the TrainedMinusT6 model-predicted window. This means, that though the TFBSs in the homologous region can be better aligned to the ones from the training region in *D. melanogaster*, the predicted output is worse than the one mediated by the predicted window, whose TFBSs can not be aligned very well to the MSE2 in *D. melanogaster*. Which of the positions is the true MSE2 for *D. ananassae *can only be determined in wet-lab experiments.

The TFBS-maps for the predicted regions and UCSC aligned MSE2s as well as a multiple alignment are shown in the Supplementary material (Additional file 2, 3 and 4).

Fig. 5b illustrates how well the predicted transcriptional response mediated by the optimal windows fit the observed mRNA concentrations. This is particular surprising for the optimal windows that do not overlap the MSE2 (exemplified for *D. mojavensis*). If not an artifact, they might be additional MSE2 regions. Small et al. [15] found that regions outside the MSE2 aid in increasing the expression level of stripe 2. Their observation was limited to regions within 8 kb upstream of *eve*. However, it may be that there are additional enhancers within the 20 kb *eve *locus.

### 0.4 Predicting the exact location of MSE2 in *D. melanogaster*

**Figure 6:**
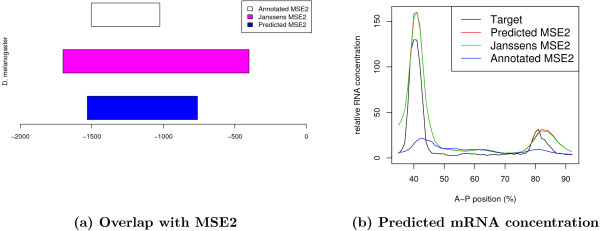
**Location and expression prediction of the MSE2 in *D. melanogaster***. Panel **a**: Overlap between the best TrainedMinusT6 model-predicted MSE2, the MSE2 region proposed by Janssens et al. [9] and the annotated MSE2 [15]. The predicted MSE2 was identified as the window with the lowest RMS error amongst all positions and window sizes. Panel **b**: Predicted mRNA concentration using the MSE2 proposed by Janssens et al. [9], the predicted MSE2 location and the annotated MSE2 location, respectively.

As part of the previous experiment, we evaluate the prediction of the best TrainedMinusT6 model using TFBS-maps corresponding to all windows of various sizes in the 20 kb surrounding *eve*. This detects a window near *eve *in *D. melanogaster *with the same RMS error as the training window, but 500 bp shorter. Fig. 6a shows the location of the shorter window relative to the 1300 bp long training window.

The 1300 bp of the extended MSE2 region used for training was constructed by Janssens *et al*. [9] to demonstrate that the sequence downstream of the annotated MSE2 has regulatory function and can mediate a weak stripe 7 expression. The extended MSE2 has, therefore, a strong stripe 2 and weak stripe 7 expression in contrast to the annotated MSE2, which only mediates the stripe 2 expression [15]. Since there was no wet-lab experiment undertaken to identify the minimal region able to evoke stripe 2 and stripe 7 expression, it is likely that the 1300 bp construct contains sequence parts with no regulatory function. The shorter (700 bp long) window identified *in-silico *may therefore be the minimal sequence element evoking the observed pattern, especially because it fully contains MSE2 and extends the region only a short stretch downstream.

The predicted outputs of the three regions are shown in Fig. 6b. While the predicted window and the training window produce the same output, the predicted response for the annotated MSE2 is very low. But, as expected, there is only a transcriptional response predicted for stripe 2, and no response at the location of stripe 7.

## Conclusion

We have introduced a computational tool to predict the transcriptional output mediated by the predicted TFBS-map of a specific CRM. We have generalized a recently developed mathematical model to make it applicable to any DNA sequence. We have demonstrated the ability of the model to predict the transcriptional response and the location of the MSE2 region in other *Drosophila *species. In six out of the eight *Drosophila *species, the model identifies the correct position as the window with the lowest RMS error. The overall low RMS error indicates that the model captures the underlying mechanism of transcriptional regulation and generalizes to control regions in other species.

However, preliminary studies (not shown) indicate that the current Reinitz model fails to generalize to CRMs other than MSE2. This is in agreement with the recent study by Segal *et al*. [14], where their model fails specifically for MSE2, but succeeds for several other CRMs in various *Drosophila *genes. This suggests that the biological mechanism involved in the regulation of MSE2 may be different than that of other enhancers. This is particularly the case for the transcription factor hb, which seems to be an activator in MSE2 while it seems to be a repressor in other enhancers [14]. How and why the regulatory mechanism can be different for MSE2 although the same set of TFs are involved remains to be investigated. Particularly, the proposed cooperativity between hb and bcd may enable hb to be an activator for the regulation of MSE2.

We further plan to substitute the biologically unrealistic discrete TFBSs with continuous binding gradients over the DNA. An interesting intermediate step would be to use TFBSs predicted by CLUSTER-BUSTER, where the reported TFBSs are not chosen based merely on their PWM scores, but by taking their location relative to other TFBSs into account.

## Methods

### 0.5 The Reinitz model

The model developed by Reinitz *et al*. [13] proposes that the control of the transcription of *eve *stripe 2 is a *deterministic *function of:

1. the concentration levels of TFs that regulate stripe 2 expression,

2. a TFBS-map, specifying the locations and "strength" of the TFBSs.

The Reinitz model computes the transcription rate of a gene regulated by a CRM, given a TFBS-map (specifying the location and log-odds score of each TFBS in a CRM), a set of concentrations of the relevant TFs and the set of free parameters (*X*_0_, *R*_0_, *E*s and *K*s). We intend to give only a brief summary of the model. For more detail, see the Supplementary material (Additional file 5) or the original publications [13,9].

*Firstly *the model estimates, for each binding site, the probability that it is occupied (bound) by its TF as a function of TF concentration, TFBS binding affinity, competition and the association constant (*K*) of the TF. *Secondly*, the model reduces the influence level (*F*) of each activator TFBS according to the number and effectiveness (*E*) of the surrounding quenchers. *Thirdly*, the model calculates the total (quenched) activation (*X*) as the sum of the influence levels of all activator sites weighted by the activator's effectiveness (*E*). The transcription rate is then computed as a function of *X*, *R*_0 _and *X*_0_,

(1) $$R=R_{0}\cdot e^{-(X_{0}-X)},$$

and artificially clipped at *R*_0 _to prevent unrealistically high transcription rates.

### 0.6 Eve mRNA expression and TF concentration data

The *eve *gene is expressed as vertical stripes in developing *D. melanogaster *embryos. The expression of *eve *in the region of the second stripe ("stripe 2"), counting stripes along the anteroposterior axis of the embryo, is known to be controlled by a CRM located about 1.7 kb upstream of the *eve *gene, illustrated in Fig. 8. This CRM is referred to in the literature as "minimal sequence element for stripe 2" (MSE2). Janssens *et al*. [9] measured the transcriptional output of a reporter gene construct consisting of the 1.7 kb region upstream of the *eve *transcription start site (TSS), including the promoter and the 5' UTR, fused to the coding region of *lacZ*. This region contains the complete MSE2, plus some additional sequence downstream of it.

Janssens *et al*. [9] generated the *eve *mRNA data by collecting embryos bearing either the full 1.7 kb upstream of the *eve *gene or just the *eve *MSE2 region (-1.5 delta -1.1 (MSE)) attached to a *lacZ *reporter gene [15]. This *eve-lacZ *fusion gene is expressed in the same way as *eve *stripe 2. The mRNA of the *lacZ *gene is visualized using *in situ *hybridization and antibody staining. The mRNA concentration is proportional to the transcription rate, because the half life of the *eve-lacZ *fusion gene-mRNA is very short (6 minutes) and can therefore not accumulate over the measured time.

**Figure 7:**
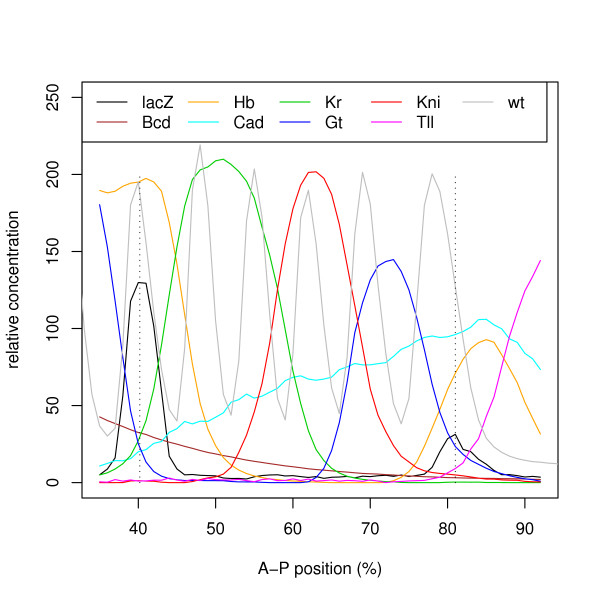
**TF protein concentration and mRNA concentration of the *eve-lacZ *fusion gene at time-point *T6***. Dotted lines give the reference point where the mRNA concentration reaches the highest point of the peak. The grey curve is the mRNA concentration of *eve *in the wild-type.

Jaeger *et al*. [8] generated the TF protein concentration data by fluorescently staining *D. melanogaster *blastoderm stage embryos for the TF proteins using antibodies. The embryos were then laterally oriented and processed with an image segmentation method to obtain the average pixel value within each nucleolus. These values were then grouped into 100 equally sized bins according to their position along the anteroposterior axis. The values in each bin were averaged and normalized to range from 0 to 255 and are defined to be the protein concentration of the TFs. To obtain the final value, the concentration of at least 10 embryos were averaged for each time point. As an example, the complete data for time point *T6 *is shown in Fig. 7.

**Figure 8:**
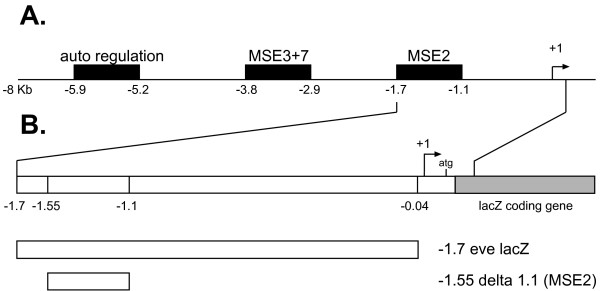
**Summary of *eve-lacZ *fusion gene**. Panel **A **shows the 8 kb of *eve *5' flanking sequences. This region has been shown to contain CRMs responsible for the initiation of stripes 2, 3 and 7 (MSE2, MSE3+7), as well as an auto-regulatory element responsible for refinement and maintenance of the seven stripes during gastrulation. Panel **B **shows the two MSE2-*lacZ *fusion gene clones used for the mRNA concentration measurements. This picture was adapted from Small *et al*. [15] Fig.1.

### 0.7 TFBS-maps

For our benchmark model (Janssens model) we used the TFBS-map reported by Janssens *et al*. [9]. For all other models we used predicted TFBS-maps obtained as follows. We use log-odds position weight matrices (PWMs) for each of the seven TFs to create predicted TFBS-maps. Given a genomic region and the PWM for a TF, we use the motif scanning algorithm FIMO to predict all sites (on both DNA strands) with log-odds score above a threshold, *T*. FIMO is available as part of the Meta-MEME package (see Availability and requirements section for URL). The positions (relative to the start of the genomic region) and log-odds scores of the predicted sites constitute the TFBS-map. We build the PWMs from so-called "count matrix" motifs representing the binding specificity of each of the TFs. LOGOs showing the information content of each TF motif are shown in the supplement (Additional file 6).

To create a log-odds PWM from each count matrix, we first add a pseudo-count to each entry, and then normalize so that the sum in each column of the motif is one. We then divide entries by the background frequencies. Finally, we take the natural logarithm of the entry. We use a log-odds score threshold of 9 bits. We use the same background frequency model as Janssens *et al*. [9], which is the base frequency of the *D. melanogaster *genome: *B*_*A *_= *B*_*T *_= 0.297 and *B*_*C *_= *B*_*G *_= 0.203. We also use this background when predicting TFBSs in other species because the PWM score contributes to the learned parameters. The protocol to provide the PWM scores should therefore not be changed. As pseudo-count we used 1.5 times the background frequency. This value was suggested by Frith *et al*. [7] to maximize the log likelihood of finding a true positive site in an empirical study with TRANSFAC [11] matrices and sites.

### 0.8 Application of the model

We applied the simulated annealing (SA) algorithm used in Janssens *et al*. [9] to train the model. This program was kindly provided to us by John Reinitz. Except for the benchmark case with the TFBS-map from Janssens *et al*. [9], we always run the SA algorithm with the only free parameters being one association constant, *K*, and effectiveness constant, *E*, for each TF. We also inactivated the so-called "direct repression coefficient" parameters (not mentioned in Janssens *et al*. [9] but present in their program). To use the model in a predictive mode, we use a program named UNFOLD, also provided to us by John Reinitz. This program takes the TFBS-map, the TF concentrations and the trained model parameters as input, and outputs the predicted mRNA concentration, *R*, for each set of TF concentrations in the input.

### 0.9 Training and validation of the model

In our experiments, we train the Reinitz model using the mRNA levels of *eve-lacZ *fusion gene TF concentrations reported by Janssens *et al*. [9], and TFBS-maps obtained from the DNA sequence surrounding *eve*. The free model parameters are optimized by the training algorithm (SA) to reduce the RMS error between the known transcription rate and the rate predicted by the model, averaged over all input points. Each data point corresponds to a particular time point in the development of a *D. melanogaster *embryo and a particular location on its anteroposterior axis (see Fig. 7). The time points are labeled *C13*, *T1*–*T6 *and the locations of interest along the anteroposterior axis are from 35% to 92%. We emphasize once more that the model is purely static, and has neither concept of time nor of position within the embryo.

To test the ability of the trained model to generalize to unseen data, we perform a seven fold cross-validation with 30 repeats. We divide the data set according to time points into seven subsets. A model is trained on all but one of the seven subsets. This is repeated holding out each of the seven time points exactly once. Because the training algorithm (SA) is stochastic, we repeat the training and testing in each fold multiple times by choosing ten random values for the starting points of the free parameters and starting SA for each set three times using different seeds. The average RMS error of the 30 models and all training time points is referred to as "training error" and the average RMS error of the models on the hold-out time-point is herein referred to as "test error".

The redundancy in the data was reduced as follows. Each data point, *X*_*i*, *j*_, is an eight dimensional vector, containing the protein concentration levels of the seven TFs and the mRNA concentration of the *lacZ*-fusion gene, at time point *i *and anteroposterior position *j *∈ *AP*. The RMS distance between two time points was calculated as

(2) $$D(t_{i},t_{2})=\sqrt{\frac{1}{\left|AP\right|}\sum_{j\in AP}{\left(X_{t_{1},j}-X_{t_{2},j}\right)}^{2}}$$

As shown in Tab. 2, the distance between *T5 *and *T6 *is the smallest. To avoid redundancy in the data set, time point *T6 *was removed because its average RMS distance to all other time points was smaller than the one from *T5*.

**Table 2:** Pairwise distance between the mRNA and TF concentration of adjacent time points.

*Pair*	*pairwise distance*
T5, T6	158
T4, T5	237
T1, T2	284
T2, T3	304
T3, T4	304
C13, T1	414

The table shows the pairwise distance as the sum of the RMS distances between the mRNA concentrations and TF concentrations of two time-points. Only adjacent time points are shown; non-adjacent time points have much greater distances.

### 0.10 Identifying the MSE2 position in other *Drosophila *species

The MSE2 region is known in *D. melanogaster *and *D. pseudoobscura *but, to the best of our knowledge, there is no annotation for the other six species. To identify the MSE2-homologue regions in other species we used the UCSC alignments. We first extracted the UCSC sequences aligned via MULTIZ [3] to the sequence of the 1300 bp of the MSE2 region in *D. melanogaster *and aligned them then to the 20 kb surrounding the *eve *gene in each species using ClustalW [6] to identify putative MSE2 regions.

According to Ludwig *et al*. [10] the similarity of the TFBS-maps between *D. melanogaster *and *D. pseudoobscura *is low due to missing and rearranged TFBSs [4]. To measure how similar the TFBSs of other species are, we aligned the TFBS-map of the putative MSE2 region of each species with the TFBS-map of *D. melanogaster *using MMETA[4]. Mmeta aligns, non-colinearly, the TFBSs between two or more TFBS-maps. It does so by optimizing the mismatches of aligned labels (TF names) and their positions in the primary sequence. It returns a similarity score representing the quality of the achieved alignment and the number of aligned TFBSs. The score is therefore unbounded, with small or negative values representing many unaligned or poorly aligned TFBSs, and with large positive values indicating alignments of identical maps.

### 0.11 Locating the MSE2 region in other species

To test the specificity of the model, we apply the model to the predicted TFBS-map of all windows surrounding *eve*. The algorithm takes a trained model, a set of data points (TF concentrations and target mRNA levels), a set of log-odds PWMs for the TFs in the model, and a genomic region as input. The algorithm computes the RMS error of windows (contiguous sub-regions of DNA) as follows. The PWMs are first used to compute a predicted TFBS-map for the window. The RMS error of the input model using the predicted TFBS-map is then computed using the input set of data points. The algorithm repeats this process for all windows of a given size and spacing along the input genomic region. We calculated the RMS error for windows spaced 10 bp apart, starting at the 5' end of the DNA surrounding *eve*. To determine the optimum window size, we try windows with widths in the range *w *= [100,..., 2000] bp in steps of 100 bp. We use the same log-odds PWMs in training and CRM prediction. In particular, we use the background model, B, for *D. melanogaster*, even when predicting CRMs in other organisms. We do this because changing the background model would change the log-odds scores of sites, and predicted site association constants, *K*_*s*_, would then be different for identical sites in different organisms.

To measure the specificity of the model predictions, we compute the PC as defined by Pevzner *et al*. [12]. For a given RMS threshold, we define

(3) $$PC(r)=\frac{\left|K\cap P(r)\right|}{\left|K\cup P(r)\right|},$$

where *K *are the "known" genomic regions in the MSE2 (annotated or alignment-identified) and *P*(*r*) are the "predicted" genomic regions predicted by the model using an RMS threshold of *r*. (The vertical bars in the notation indicate the size of the regions in base pairs). A base-pair is considered as predicted if it is part of a "minimal" window whose RMS error is equal or below the threshold *r*. A window is only "minimal" if there is no other, smaller window contained within it, whose RMS error is equal or better.

## Availability and requirements

Meta-MEME: 

## Abbreviations

*eve*: Even-skipped gene; *D. mel*: *D. melanogaster*; *D. pse*:  *D. pseudoobscura*; *D. an: D. ananassae*. *D. moj: D. mojavensis*; *D. wil*:  *D. willistoni*; *D. gri*: *D. grimshawi*. *D. vir*: *D. virilis*; *D. per*:  *D. persimilis*.

## Authors' contributions

DCB researched and carried out the experimental work under the supervision of TLB. The initial manuscript draft was written by DCB, and refined by TLB.

## Supplementary Material

Additional file 1 **Comparison of parameters from different models**. Table containing the parameters for the TFs after training was performed. Click here for file

Additional file 2 **Comparison of TFBS-maps in predicted MSE2 regions**. Table containing a visualization of the TFBS-maps from the predicted MSE2 regions using our predictor. Click here for file

Additional file 3 **Comparison of TFBS-maps in homologous MSE2 regions**. Table containing a visualization of the TFBS-maps from homologous MSE2 regions. Click here for file

Additional file 4 **Sequence conservation of homologous MSE2s**. Multiple alignment of the homologous MSE2 region in the eight different *Drosophila *species. Click here for file

Additional file 5 **The Reinitz model and PWMs**. Additional detail on the Reinitz model. Click here for file

Additional file 6 **Count matrices and logos of the used TFs**. Table containing the count matrices and logos of the TFs used in this study. Click here for file
