# Supplementary material for: Studying the functional conservation of cis-regulatory modules and their transcriptional output
Source: BMC Bioinformatics. 2008 Apr 29;9:220. doi: 10.1186/1471-2105-9-220 (PMC2386823; doi:10.1186/1471-2105-9-220)
Supplement: Additional file 1 — Comparison of parameters from different models. Table containing the parameters for the TFs after training was performed. [file 1471-2105-9-220-S1.pdf]

Additional file 1 — Comparison of parameters from different models

		TrainedAll model		*TrainedMinusT6 model*	
TF	*parameter*	*value*		*value*	
name		*average*	*(SD)*	*average*	*(SD)*
bcd	K	0.04	10^{-04}	0.03	0.01
	E	0.83	0.07	0.96	0.04
cad	K	0.01	10^{-04}	10^{-03}	10^{-04}
	E	4.24	0.80	4.64	0.81
gt	K	10^{-03}	10^{-05}	10^{-03}	10^{-05}
	E	1.00	10^{-09}	1.00	10^{-09}
hb	K	10^{-04}	10^{-05}	10^{-04}	10^{-05}
	E	10.00	10^{-07}	10.00	10^{-07}
kni	K	0.01	10^{-03}	0.01	10^{-03}
	E	1.00	10^{-08}	1.00	10^{-08}
kr	K	10^{-04}	10^{-04}	10^{-04}	10^{-04}
	E	0.94	0.18	0.83	0.27
tll	K	0.05	10^{-17}	0.04	0.01
	E	0.98	0.01	0.98	0.03
Training error		9.66	10^{-03}	9.00	0.02

Table 1: **Variance in parameter settings for the TrainedAll model and TrainedMinusT6 model.** Variability in optimal parameter values from 30 independent training runs (different starting values and seeds for simulated annealing).
