# Supplementary material for: Studying the functional conservation of cis-regulatory modules and their transcriptional output
Source: BMC Bioinformatics. 2008 Apr 29;9:220. doi: 10.1186/1471-2105-9-220 (PMC2386823; doi:10.1186/1471-2105-9-220)
Supplement: Additional file 2 — Comparison of TFBS-maps in predicted MSE2 regions. Table containing a visualization of the TFBS-maps from the predicted MSE2 regions using our predictor. [file 1471-2105-9-220-S2.pdf]

Additional file 2 — Comparison of TFBS-maps in predicted MSE2 regions

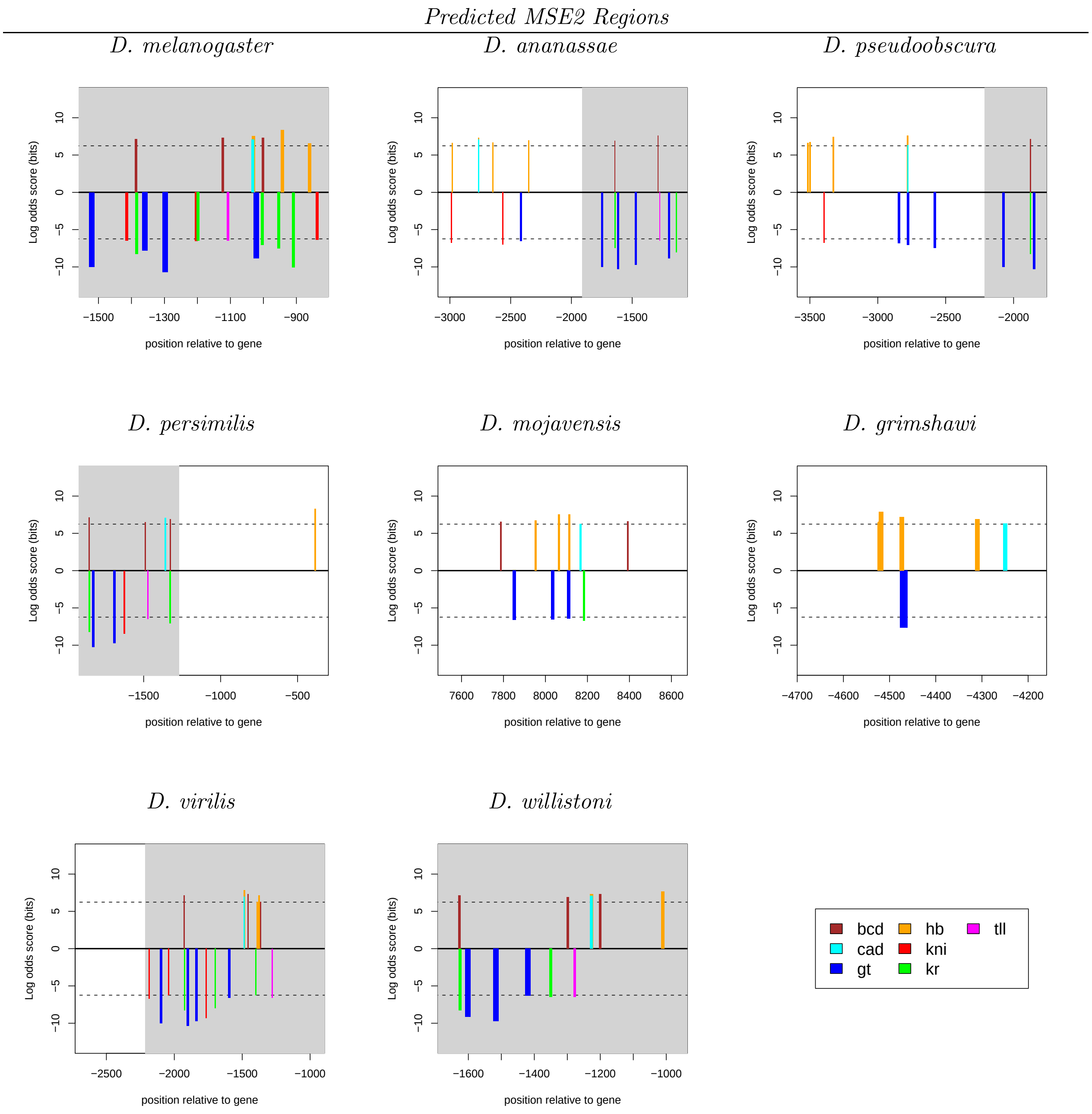

Figure 1: **Comparison of the TFBS maps in the predicted MSE2 regions in other *Drosophila* species.** The maps are generated for the sequence of each species, with the lowest RMS error. FIMO was used with *D. melanogaster* background 1.5 pseudo-count and a threshold of 9 bits. TFBSs of TFs with repressing function are drawn as negative impulses. The grey shaded area is the MSE2 region as proposed by Janssens *et al.* [10] or its homologous according to the UCSC alignment.
