# Supplementary material for: Studying the functional conservation of cis-regulatory modules and their transcriptional output
Source: BMC Bioinformatics. 2008 Apr 29;9:220. doi: 10.1186/1471-2105-9-220 (PMC2386823; doi:10.1186/1471-2105-9-220)
Supplement: Additional file 3 — Comparison of TFBS-maps in homologous MSE2 regions. Table containing a visualization of the TFBS-maps from homologous MSE2 regions. [file 1471-2105-9-220-S3.pdf]

Additional file 3 — Comparison of TFBS-maps in homologous MSE2 regions

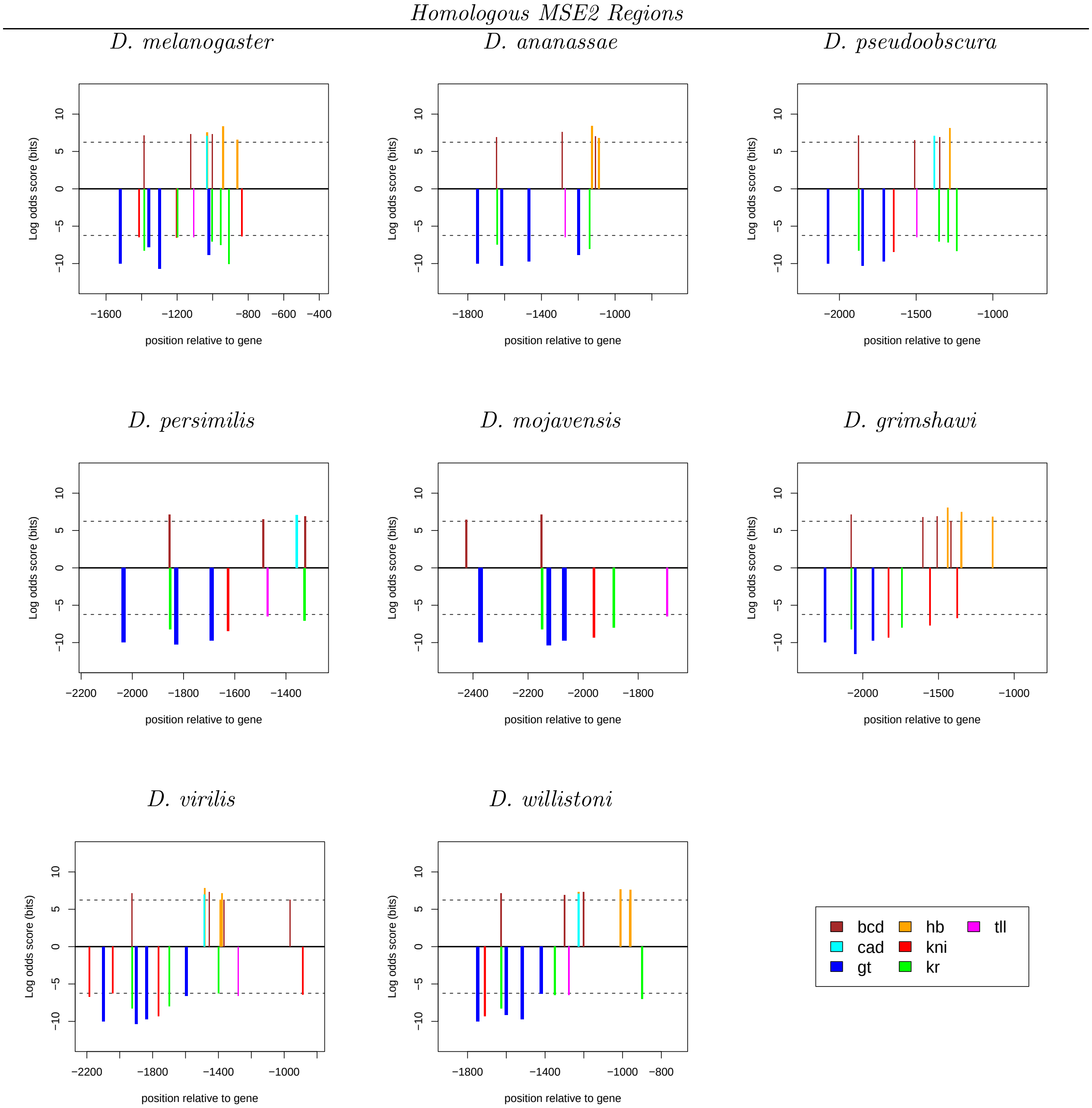

Figure 1: **Comparison of the TFBS maps in the homologous MSE2 regions in other** ***Drosophila*** **species.** The maps are generated for the sequence of each species, which was aligned (UCSC) with the 1300 bp MSE2 construct from *D. melanogaster* used by Janssens et al. [10]. FIMO was used with *D. melanogaster* background 1.5 pseudo-count and a threshold of 9 bits. TFBSs of TFs with repressing function are drawn as negative impulses.
