# Supplementary material for: Studying the functional conservation of cis-regulatory modules and their transcriptional output
Source: BMC Bioinformatics. 2008 Apr 29;9:220. doi: 10.1186/1471-2105-9-220 (PMC2386823; doi:10.1186/1471-2105-9-220)
Supplement: Additional file 5 — The Reinitz model and PWMs. Additional detail on the Reinitz model. [file 1471-2105-9-220-S5.pdf]

Additional file 5 — The Reinitz model and PWMs

The model of transcriptional regulation has been described in detail by Reinitz et al. [15]. Here, we intend only to give a general overview of the mathematical representation of the underlying biological mechanisms believed to be regulating transcription.

The general assumption is that transcription is an enzymatic process where DNA binding events of activators cause an exponential increase in transcription rate R (up to a predefined maximum R_0). The functionality of activators can be cumulatively reduced by binding events of repressors that occur in close proximity to the activator TFBSs. Whether an activator or quencher site s is occupied is modeled by a probability function which is dependent on a) the ability of the TF to bind DNA, K_t, b) the quality of the transcription factor binding site k^{s_i}, and c) by the concentration of the TF v_t. Each TF t from the set of TFs T belongs to the group of activators A or repressors, called quenchers, Q and is therefore called a or q.

We now can formalize the input data to the model as follows. The input data, D, contains a set of n pairs, $(\mathbf{V}, v_l)$, where each pair contains a vector, $\mathbf{V} = (v_1, v_2, \ldots, v_n)$ listing the concentrations of all TFs, and v_l, the observed concentration of the target gene, here lacZ mRNA concentration. The TFBSs of a TF t, that match t's PWM with a particular log-odds threshold in the DNA region r, e.g. MSE2, is represented as $S^t = \{s_i = (t, j, k, s) | i = 1, \ldots, n_t\}$. Each of the n_t sites in S^t is defined by a quadruple of TF name t, start j and end k of the TFBS and its PWM score s. Where s is the log-odds score represented to the basis of e.

The parameters Θ learned by the model contains two parameters per TF, which are the efficiency value C_a, E_q respectively, and the binding affinity K_t. Θ also contains the maximal transcription rate R_0 and the energy barrier X_0 that need to be crossed for the transcription to take place.

The output of the model is a vector of predicted mRNA concentrations, R, corresponding to each data point in $\mathbf{V}$and is defined as follows.

$$\begin{aligned} R(\mathbf{V}, S, \Theta) &= R_0 exp(-(X_0 - \gamma^Q M(\mathbf{V}, S, \Theta))) \\ &= R_0 exp(\gamma^Q M(\mathbf{V}, S, \Theta) - X_0). \end{aligned} \tag{4}$$

The function M describes the predicted, effective number of bound activators. γ^Q is a constant which defines how much the energy barrier X_0 is reduced by M, here $Q = 1$.

M is defined in terms of the constant, $\gamma^{AF} = 0.99$, and function, N (Section 6), the number of sites that are available to recruit activators.

$$M(\mathbf{X}, S, \Theta) = \gamma^{AF} N(\mathbf{X}, S, \Theta). \tag{5}$$

The amount of activators recruited by activator sites is modeled by function N. For each activator, $a \in A$, it sums the occupancy of all activator sites, after correcting for quenching, $F^A(a, i, v_a)$, and weights them by a's effectiveness parameter, C_a. The recruitment function is

$$N(\mathbf{V}, S, \Theta) = \sum_{s \in S^A} C_a F(a, i, v_a), \tag{6}$$

where, in each term in the sum, a and i are determined from the site current site, $s = (a, i)$.

The fractional occupancy of activator a at site i after quenching is modeled as the product of the unquenched occupancy function, f, multiplied by the quenching function, G. The quenched occupancy function is

$$F(a, i, v_a) = f(a, i, v_a) G^{(1, N^q)}(i). \tag{7}$$

The quencher sites, S^q, are arbitrarily ordered from one to N^q. The effect of quenching sites depends on their occupancy, f, scaled by a distance-based quenching function, $d(i, j)$, that depends on the distance between the position of the activator site, i, and the position of the quencher site, k. This, in turn, is scaled

by the effectiveness factor, E_q. (The position and TF for site s_j are determined from the site itself, since $s_j = (b, k)$.)

We define the complete quenching function using the partial quenching function, which captures the effects of quencher sites s_m through s_n, on any activator site at position i, and is given by

$$G^{(m,n)}(i) = \prod_{j=m}^{n} [1 - (d(i,k) E_q f(q,k,v_q))], \tag{8}$$

where k and q are determined for each j in the product from the values in site $s_j = (q, k)$. The complete quenching function considers the effects of all quencher sites, and is given by $G^{(1,N^Q)}(i)$.

Whether an activator or quencher site $s = (t, i)$ is occupied by the TF t is modeled as a probability function of a) the ability of the TF to bind to the site s_i, b) by the concentration of t, v_t and c) if competition for s by other TFs $t_2 \cdot t_j$.

$$f(t,i,v_t) = \frac{K(t,i)v_t}{1 + K(t,i)v_t + \sum_{j \in O_{K(t,i)}} K(t_2,j)v_b}, \tag{9}$$

where $K(t, i)$ is the binding affinity of TF t at position i in the enhancer sequence. $O_{K(t,i)}$ are all sites overlapping with $K(t, i)$ from T with $t_2 \neq t$.

The binding affinity of TF t to a site in the enhancer region is assumed to be proportional to the exponent of the difference score of the site and the maximum possible score, $\hat{S}_t$. So the binding affinity function is

$$K(s,t) = K_t exp(S(t,i) - \hat{S}_t). \tag{10}$$

The score, $S(t, i)$, is the PWM score of the site of TF t at position i in the enhancer sequence as given by

$$S(t,i) = ln\left(\frac{Pr(s_i|P)}{Pr(s_i|B)}\right) log_e(2). \tag{11}$$

where the normal log-odds score, with P as the PWM and B as the background model, is converted from bits to nats. For simplification the largest $S(t, i)$ in the input data is chosen to be the $\hat{S}_t$.

To determine the error between predicted R and observed transcription rate v_l, we define an error function as the root mean squared over the set of n observed points, $\{(\mathbf{V}, v_l)\}$ as follows,

$$E(D,\Theta) = \frac{1}{n} \sum_{\{(\mathbf{V},v_l)\}} \left(\frac{1}{2}(v_t - R(\mathbf{V},S,\Theta))^2 + P(\mathbf{V},S,\Theta)\right). \tag{12}$$
