# Supplementary material for: Studying the functional conservation of cis-regulatory modules and their transcriptional output
Source: BMC Bioinformatics. 2008 Apr 29;9:220. doi: 10.1186/1471-2105-9-220 (PMC2386823; doi:10.1186/1471-2105-9-220)
Supplement: Additional file 6 — Count matrices and logos of the used TFs. Table containing the count matrices and logos of the TFs used in this study. [file 1471-2105-9-220-S6.pdf]

Additional file 6 — Count matrices and logos of the used TFs

<table>
<tr><th>name</th><th>Count Matrix</th><th>logo</th><th>source</th></tr>
<tr><td>bcd</td><td>A C G T
9 19 5 18
11 3 1 36
49 0 2 0
51 0 0 0
0 0 17 34
1 45 0 5
1 25 4 21
4 16 21 10</td><td>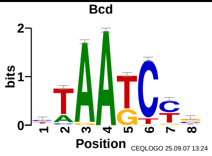</td><td>Berman et al. [2]</td></tr>
<tr><td>hb</td><td>A C G T
12 12 10 59
0 0 0 93
1 4 2 86
0 0 0 93
0 0 0 93
0 1 0 92
49 9 26 9
17 17 12 47
2 18 45 28
27 25 24 17
9 26 28 30</td><td>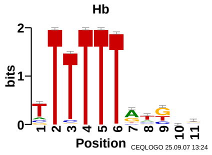</td><td>Berman et al. [2]</td></tr>
<tr><td>cad</td><td>A C G T
4 1 2 2
4 2 5 4
8 7 7 12
5 18 4 7
14 7 7 6
0 7 3 24
34 0 0 0
34 0 0 0
34 0 0 0
17 3 8 6</td><td>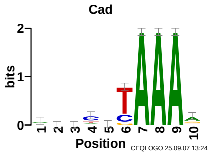</td><td>Berman et al. [2]</td></tr>
<tr><td>kr</td><td>A C G T
16 4 7 2
27 1 1 0
25 3 0 1
15 7 3 4
0 0 28 1
3 0 26 0
5 2 22 0
0 0 1 28
1 3 4 21
22 1 3 3</td><td>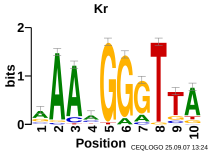</td><td>Berman et al. [2]</td></tr>
<tr><td>gt</td><td>A C G T
16 4 7 2
27 1 1 0
25 3 0 1
15 7 3 4
0 0 28 1
3 0 26 0
5 2 22 0
0 0 1 28
1 3 4 21
22 1 3 3</td><td>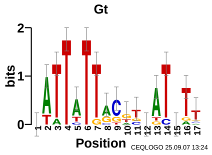</td><td>Markeev et al. [12]</td></tr>
<tr><td>kni</td><td>A C G T
5 0 0 0
5 0 0 0
1 3 1 0
0 0 2 3
4 1 0 0
0 0 5 0
3 1 1 0
0 1 2 2
0 5 0 0
5 0 0 0</td><td>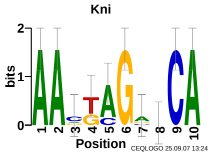</td><td>Berman et al. [2]</td></tr>
<tr><td>tll</td><td>count matrix subject to copyright</td><td>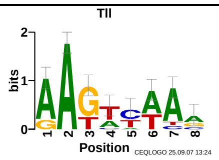</td><td>TRANSFAC [13]</td></tr>
</table>

Table 1: Count matrices and logos for the seven TFs along with the source
